# The Colon of the Horse—Its Diseases and Derangements

**Published:** 1888-01

**Authors:** Thomas Walley

**Affiliations:** Principal Royal (Dick) Veterinary College, Edinburgh


THE JOURNAL

OF

COMPARATIVE MEDICINE AND SURGERY.

VOL. IX. JANUARY, 1888. No. 1.

### ORIGINAL COMMUNICATIONS.

### ART. I.—THE COLON OF THE HORSE—ITS DISEASES AND DERANGEMENTS.

BY THOMAS WALLEY, M. R. C. V. S.
*Principal Royal (Dick) Veterinary College, Edinburgh.*

*Continued from page 363 Vol. VIII.*

### SUB-ACUTE COLIC INDIGESTION OR IMPACTION WITH DRY FOOD.

The term sub-acute is used here simply for the purpose of differentiating between those cases of indigestion produced by succulent and readily fermentable foods, and those which result from the ingestion of inordinate quantities of dry and indigestible material, *e. g.*, over-dried hay of poor quality, wheat or oat chaff, bean or pea straw, the husks of oats (meal seeds, *Scotland*) or maize.

The pre-disposing causes are the same as those which are in operation to produce acute indigestion, but there are marked differences in the symptoms and course of the two classes of cases.

*Symptoms.*—On the whole, these are less violent and less alarming than in acute indigestion. The abdominal pain is more subdued and more intermittent in its character, the pulse not so much disturbed, and the temperature is frequently not much above normal; the respiration is more or less labored, and the mucous membranes somewhat injected; in the less pronounced cases the animal may eat in the intervals of relief from pain, and for some hours fæcal matter (usually hard and dry and in the form of pellets) may be evacuated. Several days may pass by without the occurrence of important or alarming symptoms; on the contrary, the animal may be very violent from the outset and during the course of the case indications, common to the various forms of displacement, may appear and increase the anxiety of the practitioner, and the alarm of the client. Not infrequently, too, the condition of the pulse and of the mucous membranes is such as to point to the existence of enteritis, which lesion, I may remark, is slow to arise.

In very many cases which have come under my observation a series of symptoms have been presented of a character calculated to mislead and to puzzle the young practitioner or even older men, if they have not previously seen them, or, if having seen them, they have not rightly interpreted them. I refer to a train of symptoms which frequently lead to a diagnosis of bladder derangement, and in quite a number of cases I have seen young men rush away for the purpose of procuring a urinary catheter, under the impression that their patient was suffering from cystic retention. The symptoms referred to are frequent attempts at micturition, small quantities of urine being sometimes passed, and when the hand is introduced into the rectum, the presence of a tumour in the pelvic cavity. The exercise of a little common sense and a moderate acquaintance with topographical anatomy will enable the practitioner to determine the nature of the case in a very short space of time.

The pelvic tumor, if sub-acute indigestion, has usually a doughy feel and its fundus is presented towards the hand of the explorer; if gas is present it is resilient; if semi-fluid (rarely) matter, fluctuating. Straining may be induced by the presence of the hand in the rectum. Even where pelvic indications of colic impaction are absent, the explorer will generally detect the presence of large masses of ingesta, more or less hard in consistence, in the large colon. If fæcal matter is present in the rectum it is, as a rule, hard and dry, and adheres in small particles to the mucous membrane.

I have already stated that cases of this kind may go on for a period of several days. I have seen them extend from two to six, eight and even ten days, finally terminating in recovery. I remember one instance in which the case had continued for a period of eight days, and the owner, becoming wearied, had the patient destroyed without consulting me, and on post-mortem examination the whole of the contents of the bowels were in a pultaceous condition.

### TREATMENT OF SUB-ACUTE COLIC INDIGESTION.

Looking at the enormous mass of material usually lodged in the colon, and its more or less paralyzed condition, the advisability, and indeed the absolute necessity of administering a powerful intestinal stimulant at the outset can scarcely be questioned. Whether it is wise to administer such a motor stimulant as eserine, or pilocarpine, or a stimulating purge—as aloes—may be a matter of opinion, and that, too, of opinion determined by the results of experience. Personally, I prefer to give a large dose of aloes, in solution, combined with ammonia as a first dose, using laxative enemata as adjuvants and, in the course of 8, 12 or 16 hours, according to the urgency of the case, following it up with a reduced dose of aloes—combined with belladonna if there is evidence of spasms, and nux vomica or strychnine if evidence of paralysis, or oil—castor oil in preference. In reference to the dose or doses of purgative medicine tolerated in these cases, it is necessary to observe that very large doses may be given with comparative impunity, and probably larger doses of aloes are tolerated in Scotland than in any other part of the Kingdom. I have frequently administered doses of this agent three or four times in excess of the ordinary dose without producing any appreciable effects on the bowels in such cases as these under consideration.

During the progress of the case, anodynes, stimulants or sedatives should be used in accordance with the therapeutic indications of the symptoms, and in the same way venesection and the application of external irritants may be had recourse to. After the expiration of twenty-four hours, persistent abdominal fomentation should be practiced.

In reference to the employment of drastic purgatives such as croton oil, it must be borne in mind that such may do harm or may be the means of effecting incalculable good, and indeed, doubt may arise in the mind of the practitioner as to the advisability of giving cathartics at all, seeing that there is the alternative practice of adopting an expectant system of treatment by keeping the animal under the influence of opiates until the bowels react. Doubtless, much may be said in favor of this system, for, after all, the practitioner has often to choose between Scylla and Charybdis and his experience must largely determine which it shall be.

I might illustrate my meaning in this respect by a personal reference:

Some years ago I suffered from periodical attacks of perityphlitis, and during the course of these attacks the alternative systems mentioned—cathartics or opiates—were discussed and practiced; the opiate system was the more grateful, the cathartic (stimulating) system the more beneficial.

The advisability of employing such direct intestinal stimulants as calabar bean and jaborandi must also be determined by experience. Of the value of the former agent I can speak with confidence; of the latter (seeing that my experience of its effects in this direction is limited) guardedly.

Eserine—or, in preference, the sulphate—is undoubtedly of great value in uncomplicated cases of colic impaction and while its use should not be too long delayed, it must be cautiously administered where there is reason to fear actual mechanical obstruction of the bowel.

In the course of from twenty minutes to an hour the administration—hypodermically—of eserine may be followed by pronounced, and sometimes by alarming effects. The peristatic action of the bowels may be aroused to such a degree as to induce loud borborygmal sounds, followed by violent expulsion of gas per anum and also of fæces; but in addition, diaphoresis and even salivation may be excessive, and there may be universal muscular tremors and great depression—necessitating the administration of a dose of brandy or some similar stimulant.

*Paracentesis.*—during the progress of such cases as those under consideration—may be advisable, and should always be practiced if circumstances seem to warrant it. During convalescence, the same general rules as were laid down in spe king of acute indigestion must be followed.

### CHRONIC COLIC INDIGESTION.

This condition can only be referred to in a general sense, as it is only a symptom of functional or organic derangement, or an expression of some persistent cause of irritation.

Functionally, I am of the opinion that hepatic derangement is one of the most important causal factors; at the same time due allowance must be made for the probable existence of masticatory and insalivatory defects, as also for weakened gastric digestion. Arrest of the colic secretions cannot, unless there is extensive organic disease, play any important part in its production. Moter paresis, whether as the result of innervation, or of atony, or fatty changes in the muscular wall, would operate powerfully in its production.

In cases of chronic colonitis from the irritation of strongyles I have found evidence of grave interstitial changes in the muscular fibres of the gut, and in three instances I discovered in *post-mortem* examination excessive dilatation, for a distance of about two feet, of the intestine; the dilated part resembling in its contour and size a large sac filled with solid material. In these cases the intestinal wall was extremely attenuated, and in two of them several shred-like ruptures existed at the most salient parts, and through the apertures so formed food had passed and set up fatal peritonitis—as a coincidence it is note-worthy that all three horses were black in color, and in each the liver was the subject of chronic tissue changes.

The irritants most usually present to give rise to indigestion are concretions, accumulations of sand or gravel (found sometimes in the cæcum also) and entozoa. Of the latter, the strongylus tetracanthus is the most important, as not only does it produce a great amount of irritation—it gives rise also to extensive organic changes, as the formation of sub-mucous tumors and abscesses ; ulceration and resulting cicatrices. The S. armatus, notwithstanding the fact of its burrowing between and penetrating the walls of the intestine, is of less importance, as it is less numerous, does not form entozoic nests, and is much more easily got rid of. Ascarides and oxyures play their part also, and I have seen the intestines of colts, in the wooded districts of North Wales, literally packed with the different species above mentioned.

Parenthetically, I may remark that I have long held the opinion that the Strongylus tetracanthus propagates within the brood tumors or nests in the intestinal walls, and one of my main reasons for forming and subsequently adhering to this opinion was the observation of the fact, that when horses once become infested with this parasite they are seldom or ever expelled permanently from the intestines, and this holds good in animals fed throughout the year on dry food, as corn, hay and bran.

It is to me inconceivable that a perfect cure is seldom wrought, if it is necessary (as is stated by some helminthologists) for the ova or embryos of this worm to be expelled from the intestine of its host for the purpose of undergoing transformation in the body of some intermediary bearer, or in moist mud. If this were the case, stall fed horses would stand but little chance of being re-infested after the evacuation of the worms and of their eggs and embryos ; and it is further a significant fact that in town stables we seldom see more than one horse in a stud infested with these worms. In country districts several or the whole of the members of a stud may become the hosts of this parasite if they are depastured in the same field.

Foreign bodies are sometimes met with in the Colon and Cacum, and one of the most remarkable cases of this kind I have ever heard of occurred in the practice of a veterinary surgeon residing at Redditch some twenty-five or twenty-six years ago. The foreign bodies in this instance were unfinished needles and unfinished fish-hooks of various sizes. Attention was first directed to the case by the horse suffering from several attacks of colic and by the formation of an abscess in the flank, from the centre of which a needle was removed by the V. S. in attendance.

The intestines were sent to the London Veterinary College (during my pupilage days) and when the cavity of the gut was laid open the hooks and needles, to the number of many hundreds were found, some loose, others adhering to or penetrating the walls of the intestine in every direction.

*The Symptoms of Chronic Colic Indigestion*, are necessarily somewhat ambiguous and variable, their character depending upon the cause. Some, however, are common to each case; these are : irregularities of the bowels, sometimes diarrhœa, sometimes constipation; appetite fickle, voracious or morbid; the animal in some instances showing a great affection for dirt, sand, etc.; periodical attacks of colic, with or without tympany ; unthriftiness and malaise, harsh dry coat and often hidebound ; the visible mucous membranes, anæmic or injected, according to the presence or absence of irritation ; the mouth natural or furred and musty ; the pulse weak and sometimes irritable ; the abdomen may be pendulous and rotund or tucked up ; and, as a rule, there is incapability to perform severe exertion.

In the case of dilatation of the colon with accumulation of ingesta, sand, or any similar substance, the mass may be felt by rectal exploration.

When associated with, or due to chronic liver disease, jaundice is usually present, especially during the exacerbations of the ordinary symptoms ; but it must not be forgotten that in chronic liver disease we have very frequently a condition of acholia and, consequently, there may be an absence of icterus.

The symptoms of verminous disease vary mainly with the species of worms inducing it. Thus in the case of the oxyurus we have the well known rectal and anal symptoms; *i. e.*, rubbing of the tail and quarters during the night, and the deposition of the eggs mixed with a muscin-like substance on the skin at the verge of the anus and on the perinæum.

In the case of the ascaris, we have usually pendulous abdomen, periodical attacks of colic, evinced particularly when the animal is at work, and not infrequently tympany arises to such an extent as to render it somewhat difficult to remove the animal, if a harness horse, from the shafts.

Strongyles make their presence known by causing great unthriftiness, emaciation and debility; the bowels are usually relaxed, and particularly so if the horse is put to severe or fast work; and if he is exposed to adverse influences, such as cold and wet, the hair becomes erect and the animal presents a miserable appearance. The mucous membranes may be heightened in color or anæmic, and it is no uncommon thing for local dropsy—as of the abdomen, hind limbs or sheath—to make its appearance. The positive sign of the existence of worms is their presence in the fæces, ascarides usually being seen in largest numbers when horses are put on grass in the spring, or on carrots in autumn and winter. In some cases worms may be detected by rectal exploration, and I have on several occasions withdrawn my hand and arm from the rectum of colts literally besmeared with strongyles and ascarides.

In reference to the presence of concretions, it may be observed that no evidence short of the presence of one or more in the fæces can be accepted as positive—the nature of the food upon which the animal has been kept affords confirmatory evidence, as do also periodical attacks of colic; and during the existence of the pains we may have the assumption of the sitting posture.

In the event of a calculus becoming impacted in the sigmoid flexure, or in the single colon or rectum, the symptoms may assume very grave characters, or they may be simply those of sub-acute indigestion or of colic displacement; death resulting if the calculus is not expelled or if it is forced back into the larger parts of the colon, from exhaustion and, occasionally, from phlegmonous enteritis—though I would here remark that the inflammation caused by impacted concretions is usually confined to the immediate vicinity or the obstructing body. In some instances the calculus of concretion may be actually felt by rectal exploration, and I have on several occasions discovered one through the walls of the intestines.

*Treatment.*—It is manifest at the outset that the line of treatment to be adopted must be largely traced by the causal evidence. Thus, if there are signs of liver derangement our treatment must be directed towards the restoration of its function; if worms are present in the intestines they must be got rid of, and soon; but in nearly every case of this kind a cathartic may be administered as an initial proceeding with advantage, or if its administration seems to be contra-indicated, a laxative may be substituted and repeated at intervals of several days. If the evidence points to functional derangement of the bowel simply, measures must be adopted for its restoration, and as a rule the daily administration of small doses of aloes, combined with nux vomica and vegetable tonics and aromatics, will be found to produce the most satisfactory results. The mineral acids are also useful, and if anæmia exists iron may be administered with the aloes.

Dieting and work should be judiciously regulated, and sudden changes of diet, or the allowance of large quantities of cold water, should be carefully guarded against.

In reference to the expulsion of worms, I suppose every practitioner has his favorite method of treatment; personally, I have found none better than the continued administration for a considerable period of iron, common salt and vegetable tonics, with an occasional dose of cathartic medicine, as aloes and calomel; or linseed oil with turpentine chloroform and oil of absinthii. If there is evidence of the presence of the entozoa in the rectum, enemas of turpentine, salt or tobacco may be employed with benefit. Horses the subject of worms should be bedded with material that they are not likely to devour, such as peat moss, sawdust or spent bark. When there is evidence of the existence of large quantities of sand or gravel, or of concretions in the large bowels, the advisability of administering powerful cathartics or intestinal stimulants may be considered. If this course of treatment is decided upon, tolerably large doses of some lubricating oil—as linseed or rape—should be first given, and oil enemata thrown into the rectum. I have frequently seen small concretions evacuated in the natural course of defæcation, and have in my possession two tolerably large phosphatic calculi, which were expelled by the action of cathartics. If there is reason to believe that a calculus has become impacted, the practitioner has one of two courses open to him: (1) to endeavor to relax the bowels by the administration of large quantities of chloral hydrate or external fomentations ; (2) to rouse the bowels to action by the use of cathartics or physostignine. If the calculus or concretion can be reached by the hand every effort should be made to remove it. Sufflation with tobacco smoke, or the persistent injection of warm water, will each facilitate its removal.

The operation of *laparo-entrotomy* has not, to my knowledge, been much practiced for the purpose of removing foreign matter from the intestines. If a certain diagnosis were possible in these cases, I consider it would be advisable as a *dernier resort*, and I have frequently suggested it, though the opportunity of carrying it out has not been afforded me.

In a paper prepared by Mr. Fred. Smith, A. V. D., for discussion at the meeting of the National Veterinary Association this year, the record of a case of *laparo-entrotomy*, performed for the purpose of removing gravel from the colon, is given. The operation was not successful, but as Mr. Smith justly observes, such operations being only performed at the last moment, a fair chance for recovery is *seldom* afforded.

### INFLAMMATION OF THE COLON—COLONITIS.

As in the case of other hollow organs the inflammatory, action may be confined to the mucous membrane only, or it may involve the muscular and even the peritoneal coat.

*Colic mucositis* may be *catarrhal-croupous*, or *diphtheritic* in character; its cause may be acute or sub-acute.

It is caused by exposure to cold, especially when an animal is over-heated, and by irritant foods or drugs, or foul water. One of the worst cases I have ever seen was produced by the ingestion of cake composed of damaged oil-cake, inferior grains, treacle and castor oil farnia, the lesions on post-mortem being closely allied to those of diphtheria.

As it is manifestly impossible for an attack of colic mucositis to arise independently of inflammation of the small bowels, it is unnecessary to discuss the subject further in this place, but for important reasons I will say a few words on the subject of *phlegmonous colonitis.* This condition may, and does arise independently of the small bowels, and also as extension of inflammation, from the mucous membranes, but while I acknowledge that this may occur, I unhesitatingly affirm that acute idiopathic colonitis of a phlegmonous character is comparatively of rare occurrence, and further that nineteen cases out of twenty of so-called acute enteritis, are not cases of acute enteritis at all ; they are cases of colic displacement, associated with partial or complete vascular strangulation.

Extended reference to this subject will be made hereafter, but I may here observe that it is now about eighteen years since I formulated this conclusion in my own mind, and I have never lost an opportunity of verifying the truth of the conclusion and of impressing it upon others.*

The main cause of my original scepticism as to the frequency of cases of acute phlegmonous enteritis was the oft repeated and generally accepted statement "that death is produced in a few hours." On seriously considering the

---

*Metastatic Colonitis*, as the result of translation of disease in laminitis and croupous pneumonia, and in the parturient mare, of metritis, is far more frequently seen than is the idiopathic form.

matter, I felt that this was, to say the least, very improbable, and subsequent observation converted my doubt into certainty. Acute enteritis does not usually kill under eighteen hours—sometimes certainly in a little less time, but in my experience more frequently longer. Many of the so-called cases of enteritis win their cause in six or eight hours—this will be dilated upon in referring to colic displacement.

*The Symptoms of Phlegmonous Colonitis* are of a grave character throughout. There is great vascular excitement, as indicated by a full, rapid and hard pulse, and this is so marked and persistent as to be but slightly affected by such powerful vascular depressants as venesection and aconite. The temperature usually ranges from 105° to 107° F., the breathing is rapid, the nostrils being widely dilated, and the mucous membranes intensely injected—bright red at the outset and a Modena red at the close of the case. The mouth is hot and dry, (in my opinion one of the most characteristic of the symptoms), constipation is marked, and if the hand is introduced into the rectum its temperature is greatly elevated, the mucous membrane dry and not infrequently the hand, when withdrawn, is smeared with blood, small scibalæ of fæces and inspissated mucous. The urine is scanty in quantity and high in color ; perspiration is profuse from the commencement to the close of the case. Enteralgia is persistent and violent, and, contrary to the usually accepted (and taught) theory, the animal is utterly careless as to how it lies down or as to what becomes of it.

There is no more desperate form of enteralgia, with the exception of that accompanying complete strangulation, than that induced by acute inflammation—the animal frequently becoming delirious and unmanageable, and inflicting serious injury upon itself and causing great damage to its surroundings. Absence of thirst and refusal of food are other diagnostic symptoms of inflammation.

In the event of resolution, the alarming symptoms gradually subside. The secretions are restored, the animal, though remaining for some hours very dull and depressed, seeks for food and water, lies down quietly, and when down rests in comparative comfort ; the bowels also react, and if tympany has existed the gas is gradually expelled from the bowels.

If the case terminates in mortification, the alarming symptoms subside very suddenly, and a false hope is created in the minds of the attendants that recovery is insured, but this hope is in the course of two or three hours dispelled. The pulse becomes weak, soft and rapid, and quickly runs down, the mucous membranes are livid, the mouth and skin deathly cold, and the latter bathed in cold, clammy perspiration ; rigors make their appearance, the temperature rapidly lowers, the breathing becomes rapid and jerking in character, and the animal frequently sighs and yawns. The ears become lopped, the eyes dull and expressionless, and the countenance gradually assumes that indescribable appearance known as Hippocratic.

The patient may die in a state of wild and uncontrollable delirium, or its life may pass away like the dying flicker of a spent taper.

Insome cases an immediately fatal issue is warded off by translation of disease to some other organ.

While *convalescence* is comparatively rapid, the system of a horse that has been the subject of an acute attack of colonitis must, necessarily, have received a severe shock, and not infrequently there is extensive bruising of the skin over the prominent bony parts of the body ; moreover, the functional activity of the bowel itself must be depressed for some considerable time, and the tissue elements of its walls weakened, hence careful management for at least ten days or a fortnight after the subsidence of the acute symptoms is requisite in every case.

*Treatment of Acute Colonitis.*—If there is one disease more than another in which the virtue of therapeutics is tested, it is in colonitis. Moreover, the practitioner must form a clear conception in his own mind of the therapeutic means to be employed and the methods of their application.

Medicinally all drugs of an irritant character must be discarded, and antiphlogistics and opiates alone administered. Of these agents, a judicious combination of opium or morphia, of belladonna, atropine and aconite, is the most reliable; potassic nitrate is an admirable adjuvant. A favorite method of treatment of the late Mr. Edward Stanley, of Birmingham, was a combination of opium and calomel in small doses, oft repeated, and this treatment has been largely practiced in human medicine.

Venesection is, in my opinion, indicated and should be repeated if necessary, during the course of the case. If practiced, a comparatively large quantity of blood should be withdrawn, in fact the depletion should be continued until a distinctly lowering effect is exerted in the pulse. Sedative opiate enemas, as of laudanum and prussic acid, are valuable and should be thrown up at comparatively short intervals. Counter-irritants are useless and indeed injurious, but an occasional application of a stimulating anodyne liniment, with persistent hot fomentations to the abdomen, will afford relief.

### COLIC RUPTURE.

Rupture of the colon is a comparatively common lesion in the horse. Its production is formed by any persisting organic weakness, and by repletion of the bowel with food and water; the actual cause is violence inflicted in rolling and falling and the eccentric pressure of gas in tympany. The site of rupture is usually at the free border of the great flexures.

*The Signs of Rupture* are rarely, if ever, diagnostic—though its existence may be feared whenever grave symptoms follow (in the course of an hour or two) the sudden subsidence of violent pain in acute indigestion and tympany.

I have frequently expressed a confident opinion as to the existence of rupture in cases that I have been watching for some time, and in which a sudden subsidence in the acute symptoms had taken place, and in which, on rectal exploration, I have detected a sudden and marked change in the physical condition of the bowel: *i. e.*, if on rectal exploration I have felt the colon packed with ingesta, and occupying the posterior part of the abdominal and the pelvic cavities, and the animal suffering much pain, and have in the course of a few hours afterwards made another examination and found that the distended bowel has disappeared, and with this disappearance there has been a sudden cessation of pain, especially if the animal has been rolling previously, I have had little hesitation in expressing a strong opinion that rupture has taken place; and in the majority of instances the results of the *post mortem* examination have confirmed the *ante-mortem* opinion.

### COLIC FLUXES—APOPLEXY AND ŒDEMA

Are both very grave, but, fortunately, not very common lesions. The causes are in some cases identical, and the line of demarcation between them is a very narrow one.

Apoplexy may be diffuse or circumscribed, *i. e.*, may involve a large area of the bowel or may be confined within very small limits; it may also be interrupted and may involve the small bowel. Œdema is usually diffuse, and involves the single colon and the rectum—rarely the small intestines. Both lesions are more frequently symptomatic than idiopathic, and as symptomatic conditions, are usually secondary. The diseases and conditions with which, in my experience, they have been associated, are influenzas (usually effusive fever, or so-called "Pink Eye"), purpura hemorrhagica, acute or inflammatory, œdema of the limbs, anthracoid and septic diseases—hydrœmia, spanœmia and hœmo-globinœmia.

I have occasionally seen cases of acute apoplexy arise secondarily to laminitis and allied affections, and even as I write a laminitic patient of my own is lying *in extremis* from intestinal apoplexy.

*The duration* of these cases may be a few hours only, or they may extend over a period of from eighteen to thirty-six, or even forty-eight hours.

*The symptoms* may be quite ambiguous and the true nature of such cases can often be determined only by *post-mortem* examination. The best guide to the existence of these lesions in hemorrhagic and effusive affections is the sudden dispersal or disappearance of external lesions, and the simultaneous appearance of enteric symptoms. The positive signs of enteric apoplexy is, of course, admixture of blood with the fæcal discharges and the development of concomitant symptoms of severe internal hemorrhage.

To assert that there is, in every case, diagnostic evidence of intestinal œdema would be wide of the truth ; but notwithstanding this, I have, on several occasions in my life, succeeded in making a correct diagnosis, and particularly so if my patient has been the subject of an attack of such diseases as those alluded to above.

Enteralgia is not violent, and on introducing the hand into the rectum the temperature is either normal or lowered ; if the rectum is involved in the lesion the thickened, doughy condition of its mucous and sub-mucous tissues will be at once distinguished. Even when the rectum is not involved the thickened state of the colon can be detected, by careful and intelligent manipulation, through its walls.

In one case which came under my notice as a complication of effusive fever, the horse frequently assumed the sitting posture, and the œdema was so marked as to interfere with the evacuation of the fæces and the introduction of the hand into the rectum.

*Treatment.* To a certain extent the treatment of these cases must be regulated by their history and the nature of the determining causes. On the whole, however, the treatment is the same in every instance. It consists of the administration of styptics combined with opiates ; and of these I have found none so reliable as turpentine and tincture of opium, given both by ingestion and by rectal injection. Cathartics are contra-indicated, but a laxative, as linseed oil, is sometimes useful. In apoplexy, tincture of hamamelis should be combined with the turpentine, and in œdema, spirits ætheris nitrici and potassic iodide. If signs of exhaustion or sinking appear, stimulants should be judiciously administered—in preference brandy in apoplexy ; gin in œdema. The abdomen may be enveloped in cold cloths. I prefer warm (not hot) applications—moderate warmth determines blood to the skin and relieves pain—cold and heat repel it.

The *post-mortem* indications of these lesions are severally diagnostic. In apoplexy, blood, either coagulated or semi-fluid, is always present in the intestines ; it has frequently, (especially in hœmal lesions) a jelly-like appearance. Actual solution of continuity of the mucous membrane may or may not exist, but in any case it is deeply stained by the coloring matter of the blood, and more or less friable ; occasionally it is gangrenous. The apoplectic lesions may be associated with those of œdema; they may be quite distinct. Where *intra-mural* hemorrhage is extensive the case may be complicated by rupture ; in fact this lesion may be the actual cause of death.

In uncomplicated œdema, the *post-mortem* lesions are characteristic. The bowel is enormously thickened and increased in size—serum is thrown out into the structure of the mucous membrane itself and into the sub-mucous tissue, the meshes of which are charged to their utmost capacity with serum, which may be colorless or of a pale straw color; the surface of the membrane is much convoluted and it is extremely friable ; the capillaries are engorged with blood. The connecting tissue at the attached border of the intestine is, usually, also charged with serum and the superficial lymphatics are distended to their utmost capacity with the same fluid. The bowel has a glistening appearance, and when incised and pressure applied, large quantities of serum escape from the incisions.

